# Using gene methylation detection in bronchoscopic samples combined with morphology-based pathology for lung cancer diagnosis

**DOI:** 10.3389/fonc.2026.1799979

**Published:** 2026-03-16

**Authors:** Xiaojuan Niu, Dixia Hu, Xia Li, Lisha Wang, Xunbo Wang

**Affiliations:** 1 Department of Respiratory and Critical Care Medicine, Zhangjiakou First Hospital, Zhangjiakou, Hebei, China; 2 Department of Clinical Laboratory, Zhangjiakou First Hospital, Zhangjiakou, Hebei, China; 3 Department of Respiratory Medicine, Zhangjiakou First Hospital, Zhangjiakou, Hebei, China; 4 Department of Clinical Laboratory, The First Hospital of Hebei Medical University, Shijiazhuang, Hebei, China; 5 Hebei Innovation Center of Clinical Medical Laboratory Technology, Shijiazhuang, Hebei, China

**Keywords:** bronchoscopy, gene methylation, lung cancer, RASSF1A, SHOX2

## Abstract

**Introduction:**

Lung cancer is a common malignant tumor with high incidence and mortality. Bronchoscopy is key for its diagnosis, but traditional morphology-based pathology has limitations like missed diagnoses. This study explored the auxiliary diagnostic value of gene methylation detection combined with morphology-based pathology in bronchoscopic samples.

**Methods:**

A retrospective analysis was conducted on 654 patients who underwent bronchoscopy at Zhangjiakou First Hospital between March 2023 and May 2024. The cohort included 571 alveolar lavage fluid samples and 83 transbronchial needle aspiration (TBNA) samples. Methylation-specific PCR (MS-PCR) was employed to detect the methylation status of the SHOX2 and RASSF1A genes in these samples. The diagnostic value was evaluated by comparing the results with the final clinical diagnosis.

**Results:**

For alveolar lavage fluid samples, the sensitivity of combined SHOX2 and RASSF1A methylation detection reached 94.1%, which was significantly higher than the 30.0% sensitivity of morphology-based pathology (P < 0.001). For TBNA needle tract washing fluid samples, the sensitivity of combined methylation detection was 94.8%, higher than the 86.2% sensitivity of morphology-based pathology, though the difference was not statistically significant (P = 0.075). When gene methylation detection was combined with morphology-based pathology, the area under the curve (AUC) values were 0.9431 for alveolar lavage fluid samples and 0.9828 for TBNA samples, indicating favorable diagnostic performance. Additionally, strong positive results from methylation detection exhibited a high positive predictive value for lung cancer.

**Discussion:**

Gene methylation detection in alveolar lavage fluid and TBNA needle tract washing fluid, when combined with morphology-based pathology, can enhance the diagnostic efficiency of lung cancer and is worthy of further research.

## Introduction

Lung cancer is the malignant tumor with the highest incidence and death rate in the world. Because China has a large population, plus the problem of social aging getting worse and environmental pollution, the number of lung cancer patients in China has always been the first in the world (1). With the popularization of low-dose spiral CT (LDCT) screening, China finds 10–20 million new lung nodule patients every year. But LDCT has obvious false positive and over-diagnosis problems (2), still need bronchoscopy or percutaneous puncture and other methods to further confirm the diagnosis. The application of bronchoscopy opens a new way for the diagnosis and treatment of clinical respiratory system diseases, improving the detection rate of cancer cells through accurate sampling under endoscope.

Patients who undergo bronchoscopy examination and diagnosis can usually be divided into three types: patients with visible endoscopic changes. These patients usually take the sampling method of bronchoscopy biopsy plus post-biopsy washing, and the sampling quality is usually high; The second is patients with invisible endoscopic changes. These patients usually take bronchoscopy distal lavage, try to obtain lesion site cells, and the sampling quality is usually poor; Third, for patients with lesions outside the bronchial wall or needing to collect lymph node samples, sampling is done through transbronchial needle aspiration (TBNA), and the sampling quality is usually high but the sample size is very limited. For the latter two types of patients, simply carrying out cytopathological or histopathological diagnosis not only has the risk of missed diagnosis, but also may waste precious TBNA materials, leading to unnecessary secondary surgery. In addition, the sample size of TBNA is usually very limited, just enough for traditional pathological diagnosis, which also makes it difficult to carry out diversified gene testing or immune target testing.

Pathological diagnosis is an important basis for clinical treatment decisions, but morphological pathological diagnosis has limitations. For patients with invisible endoscopic changes, the number and proportion of cancer cells in alveolar lavage fluid may be very low. As an empirical discipline, morphological pathological diagnosis has a high probability of missed diagnosis (3). Histopathology is the gold standard for diagnosis. TBNA sampling is accurate but the sample volume is small, especially when there are many necrotic tissues or atypical tissue differentiation in the sample, there is also a certain probability of missed diagnosis. Moreover, the price of TBNA diagnosis is high, and secondary sampling will cause greater economic pressure on patients (4). At this time, clinical practice urgently needs diagnostic methods other than traditional pathology, and issue a joint diagnosis report together with pathological diagnosis to provide more objective and sensitive diagnostic results.

Epigenetic dysregulation represented by DNA methylation is closely associated with the initiation and progression of lung cancer and various other malignancies (5–9). Multiple studies on aberrant DNA methylation in peripheral blood have demonstrated that elevated DNA methylation levels are overall correlated with an increased risk of lung cancer (OR = 1.24, 95% CI: 1.10~1.39). Elevated DNA methylation levels were significantly associated with an increased risk of lung cancer when the follow-up duration was ≤5 years (OR = 1.46, 95% CI: 1.08~1.98) and 5~10 years (OR = 1.20, 95% CI: 1.06~1.36). Peripheral blood samples offer the advantage of easy accessibility, yet as a pan-cancer biomarker, the specificity of DNA methylation becomes a major limiting factor for its application beyond the fields of early screening and follow-up monitoring. Therefore, methylation detection in pulmonary exfoliated cell samples exhibits unique advantages for auxiliary diagnosis and treatment decision-making. The combined detection of SHOX2 and RASSF1A adopted in this study has obtained marketing approval from the National Medical Products Administration (NMPA) of China and is gradually gaining recognition among clinicians in China. Among them, short stature homobox2 (SHOX2) is a bidirectional regulatory gene, which plays an important regulatory role in bone development and tumor formation (10). Ras-association domain family 1A (RASSF1A) is a widely studied tumor suppressor gene, and its high methylation is closely related to tumorigenesis (11, 12). Through methylation-specific PCR (MS-PCR), we can accurately identify the CpG islands in the promoter region of the methylated gene, and quantify the abundance of methylation signal through a fluorescence quantitative PCR instrument, providing clinical with an objective numerical test report.

This study innovatively used TBNA needle tract washing fluid for gene methylation detection. And retrospectively collected the data of patients with bronchoscopic alveolar lavage fluid and bronchoscopic TBNA samples. Taking the patient’s final clinical diagnosis as the gold standard, explore the auxiliary diagnostic effect of gene methylation test combined with traditional morphology-based pathology on bronchoscopic sampling, and try to provide more abundant diagnostic strategies for clinical practice.

## Methods

### Sample information

**Figure 1:**
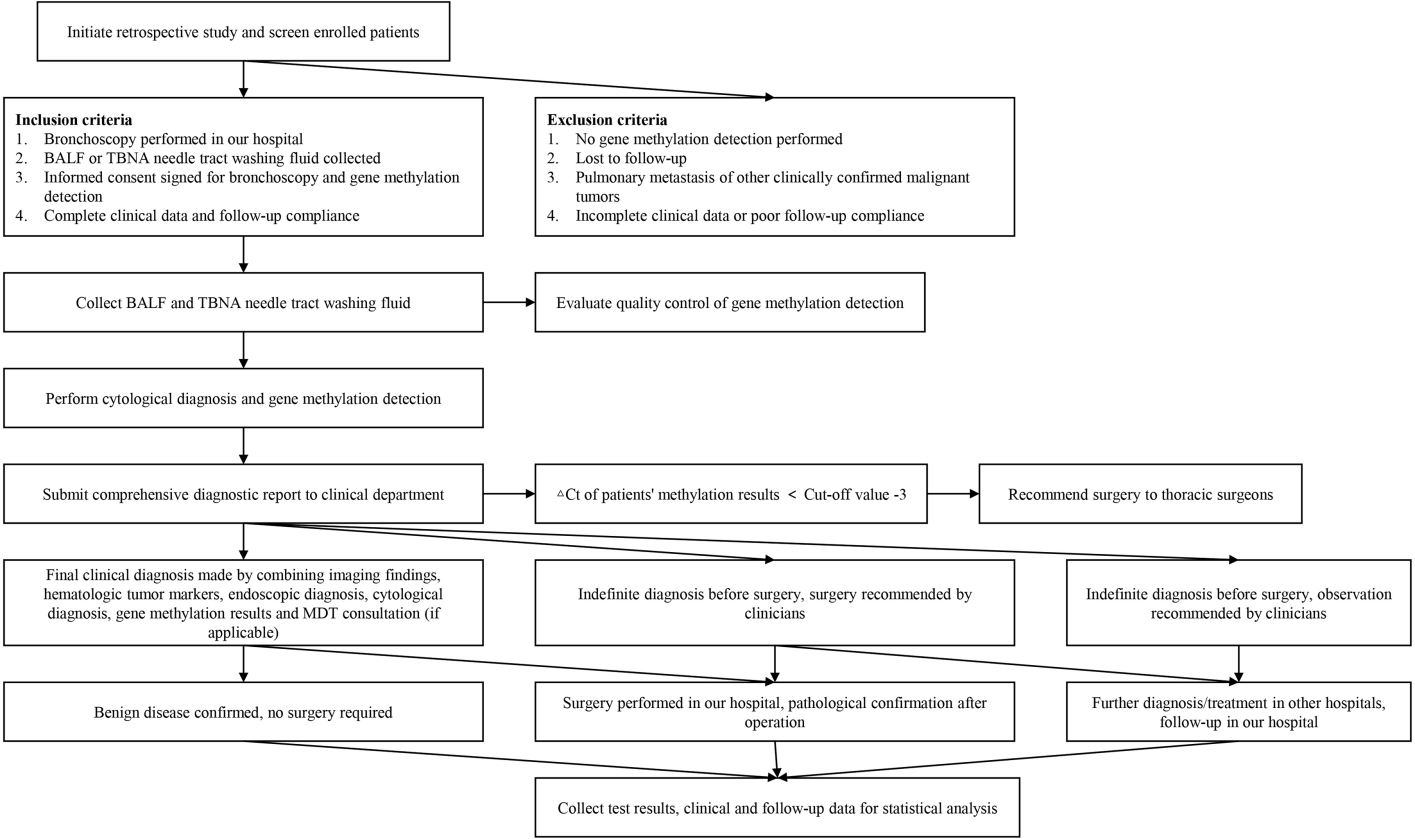
The flowchart of this study.

This study retrospectively enrolled patients who underwent bronchoscopy at Zhangjiakou First Hospital from March 2023 to May 2024. A total of 654 patients were included, with each patient providing only one type of specimen, including 571 bronchoalveolar lavage fluid (BALF) samples and 83 transbronchial needle aspiration (TBNA) samples. At the same time, information including patients’ epidemiological data, traditional morphological pathological diagnosis results, gene methylation test results, pathological stage and patients’ final clinical diagnosis were collected. All testing products used in this study are legal products approved by NMPA, and participants provided written informed consent. Patient follow-up work is undertaken by the hospital follow-up office. The flowchart of this study is shown in **Figure 1**. This study was approved by the Ethics Committee of Zhangjiakou First Hospital, with the project number 2025-LW-29.

### The patient underwent bronchoscopy and TBNA biopsy

Bronchoscopy was performed according to the summary of the 2013 British Thoracic Society guidelines for diagnostic flexible bronchoscopy in adults (13). The bronchoscope used is Olympus CV-260SL model, which can enter the main bronchus, segmental bronchus and subsegmental bronchus. If visible lesions are found under endoscope, we will first use a sampling brush to sample, and then lavage the sampling site. If no visible lesions are found, bronchoscopy is used for distal lavage. We usually use 50mL 37 °C sterile normal saline to lavage the lesion site, and then immediately use negative pressure to recover the lavage fluid. Two-thirds of the obtained alveolar lavage fluid is used for morphological pathological diagnosis, and one-third is put into a special preservation solution for methylation detection for subsequent gene methylation detection.

For patients who need bronchoscopic TBNA, according to preoperative image positioning or using ultrasound guidance, the bronchoscope is inserted into the airway, and the front section of the bronchoscope is close to the airway wall corresponding to the lesion site. Avoiding blood vessels, insert the TBNA puncture needle into the lesion, keep negative pressure and insert and extract the puncture needle 3–5 times to obtain enough cells or tissues, then release the negative pressure and withdraw the puncture needle and bronchoscope. The sample in the puncture needle is sent to the pathology department for traditional morphological pathological diagnosis, and then the puncture needle is inserted into the methylation sample preservation solution, aspirate the preservation solution 3 times, and wash the exfoliated cells in the needle tract into the preservation solution for subsequent gene methylation detection.

### Sample gene methylation detection

The methodologies employed for DNA extraction and bisulfite purification were in line with what has been documented in prior studies. The relevant kits were acquired from Beijing Jiaheng Yongtai Technology Co., Ltd (14). Subsequent to the purification process, the DNA that had undergone bisulfite conversion was subjected to amplification in parallel reaction tubes via multiple methylation-specific real-time polymerase chain reaction (MS-PCR). In this MS-PCR assay, the methylated forms of the SHOX2 gene, RASSF1A gene, and β-ACTB gene were targeted for amplification. Notably, the β-ACTB gene functioned as an internal reference to quantify the total amount of input DNA. For the detection of PCR amplicons derived from sodium bisulfite-modified sequences, TaqMan probes were utilized.

The total volume of each PCR reaction system was set at 40 μL, which comprised 5 μL of bisulfite-modified DNA template, 250 μM deoxynucleotide triphosphates (dNTPs), 0.8 μL of each primer (with a concentration of 10 μM), 1.5–3 mM magnesium chloride (MgCl_2_), 20 μL of 2×Taq buffer (this buffer already contained dNTPs and Taq polymerase), and 13.4 μL of double-distilled water (ddH_2_O). The PCR amplification was performed using a thermocycler, following a specific cycling protocol: an initial denaturation step at 95 °C for 10 minutes; 45 cycles consisting of denaturation at 95 °C for 30 seconds, annealing at a specific temperature of 58 °C for 35 seconds, and extension at 72 °C for 30 seconds; and a final extension phase at 72 °C lasting 8 minutes.

The principle underlying this detection relies on sodium bisulfite’s ability to modify unmethylated cytosine residues into uracil, which are then further converted to thymine during the PCR amplification process. In contrast, methylated cytosine residues remain unaltered through these steps, thereby allowing for the distinction between methylated and unmethylated cytosine bases. To facilitate the PCR reaction, specific primers were designed to target the DNA sequences after bisulfite modification. The detection of the resulting PCR amplification products was achieved using TaqMan probes at a concentration of 85 nM.

For quality control purposes, a plasmid containing the corresponding methylated gene sequence was used as the positive control, while purified water served as the negative control. The β-actin gene (β-ACTB) acted as the internal control to quantify the total input DNA. To obtain the cycle threshold (Ct) value of the fluorescence signal, the baseline parameters were adjusted, with the Threshold set at 10000 and the Noise band at 0.8. The Ct value corresponding to the β-actin gene signal was required to fall within the range of 18 to 32. In the context of multi-gene combined detection, given that the functions of each individual gene are mutually independent, the combined detection result was determined to be positive if any one of the target genes tested positive.

The relative methylation levels of the SHOX2 and RASSF1A genes were calculated using the delta cycle threshold (ΔCt) method. To improve the stability of the quantitative real-time PCR (qPCR) results and reduce the impact of non-specific amplification, the fluorescence signal intensity of the target gene was normalized. This normalization was accomplished by subtracting the corresponding fluorescence signal intensity of an endogenous housekeeping gene (β-actin) from that of the target gene. This step of normalization is of great significance for obtaining reliable and reproducible gene expression data, as it compensates for variations in sample quality, reverse transcription efficiency, and PCR amplification efficiency. The specific calculation formulas are as follows: ΔCt _SHOX2_ = Ct value of SHOX2 gene – Ct value of beta-actin gene; ΔCt _RASSF1A_ = Ct value of RASSF1A gene – Ct value of β-actin gene.

As a product approved by the National Medical Products Administration (NMPA) of China, the cut-off value for bronchoalveolar lavage fluid (BALF) was established based on the verification of a large number of clinical samples conducted by regulatory authorities. However, due to the lack of existing research data regarding the cut-off value for bronchoscopic transbronchial needle aspiration (TBNA) needle tract washing fluid, this study undertook the task of re-establishing this cut-off value.

### Statistical analysis

The statistical analyses in this study were conducted with the SPSS 19.0 software package, which is developed by SPSS Inc. located in Chicago, IL, USA. For the analysis of the methylation frequencies associated with the SHOX2 and RASSF1A genes, along with the results derived from bronchoscopy and morphology-based pathology, the McNemar’s test was adopted as the statistical method. To assess the diagnostic performance of the relevant indicators, the receiver operating characteristic (ROC) curve was plotted, and the area under the ROC curve (AUC) was calculated based on this curve. In the present study, statistical significance was defined as a P value less than 0.05.

## Results

### Patient characteristics

A total of 654 patients who underwent bronchoscopy were enrolled in this study, including 571 alveolar lavage fluid samples and 83 bronchoscopic TBNA samples (**Table 1**). In this study, the final clinical diagnosis of patients was used as the gold standard. For patients with a definitive diagnosis prior to surgery, the diagnostic basis was derived from clinicians’ comprehensive assessment of patients’ imaging findings, hematologic tumor markers, endoscopic diagnoses, cytological diagnoses, histopathological diagnoses, gene methylation detection results and multidisciplinary team (MDT) consultation conclusions (if applicable). For patients who underwent surgery in our hospital, the diagnostic basis was based on the postoperative pathological diagnosis as the key evidence, in addition to the aforementioned clinical information. For patients diagnosed with benign diseases in our hospital before surgery, the diagnostic evidence included, in addition to the above preoperative examination results, tuberculosis X-pert testing, PCR detection of common pathogenic microorganisms, and microbial metagenomic sequencing (if applicable). Follow-up outcomes were provided by the follow-up office of our hospital for all patients with a confirmed benign disease in our hospital or those referred to other hospitals for further diagnosis/treatment, with a median follow-up period of 18 months. Among the alveolar lavage fluid samples, 220 were finally diagnosed with lung cancer, and 351 were finally diagnosed with benign lung diseases. Among the TBNA samples, 58 were diagnosed with lung cancer and 25 with benign diseases. The average age of all enrolled patients was 63 years old, and the proportion of patients aged 60–70 years reached 43.0%, which was significantly higher than that of other age groups (2 = 61.89, P < 0.001). There were 493 male patients, accounting for 75.4%, which was significantly higher than that of females (2 = 72.36, P < 0.001).

**Table 1:** Patient demographic and clinical features.

	Total	Bronchoalveolar lavage fluid	Transbronchial needle aspiration
Age, n	654	571	83
≤50 years, n (%)	87(13.3)	79(13.8)	8(9.6)
51–60 years, n (%)	183(28.0)	159(27.9)	24(28.9)
60–70 years, n (%)	281(43.0)	250(43.8)	31(37.3)
>70 years, n (%)	103(15.7)	83(14.5)	20(24.1)
Median age	63	62	63
Age range	30-88	30-88	30-84
Gender			
Female, n (%)	161(24.6)	139(24.3)	22(26.5)
Male, n (%)	493(75.4)	432(75.7)	61(73.5)
Lung cancer, n	278	220	58
Lung adenocarcinoma, n (%)	77(27.7)	38(17.3)	39(67.2)
Lung squamous cell carcinoma, n (%)	127(45.7)	112(50.9)	15(25.9)
Small cell lung cancer, n (%)	42(15.1)	38(17.3)	4(6.9)
Unclassified lung cancer, n(%)	32(11.5)	32(14.5)	0(0)
Benign diseases, n	376	351	25
Pulmonary tuberculosis, n (%)	232(61.7)	219(62.4)	12(48.0)
Other benign lung diseases, n (%)	144(38.3)	132(37.6)	13(52.0)
Tumor stage			
Stage I, n (%)	17(6.1)	11(5.9)	6(10.3)
Stage II, n (%)	31(11.2)	27(14.4)	4(6.9)
Stage III, n (%)	88(31.7)	73(38.8)	15(25.9)
Stage IV, n (%)	110(39.6)	77(41.0)	33(56.9)

Among the 220 cases of lung cancer patients’ alveolar lavage fluid, the proportions of lung adenocarcinoma, lung squamous cell carcinoma, small cell lung cancer and unclassified lung cancer were 17.3%, 50.9%, 17.3% and 14.5% respectively. Among the 58 lung cancer TBNA samples, the proportions of lung adenocarcinoma, lung squamous cell carcinoma and small cell lung cancer were 67.2%, 25.9% and 6.9% respectively. Among the enrolled patients with benign diseases, 38.3% were pulmonary tuberculosis, and 61.7% were other benign lung diseases. Among all lung cancer patients, 6.1% were in stage I, 11.2% in stage II, 31.7% in stage III, and 39.6% in stage IV.

### Quality control of methylation detection in TBNA needle tract washing fluid

**Figure 2:**
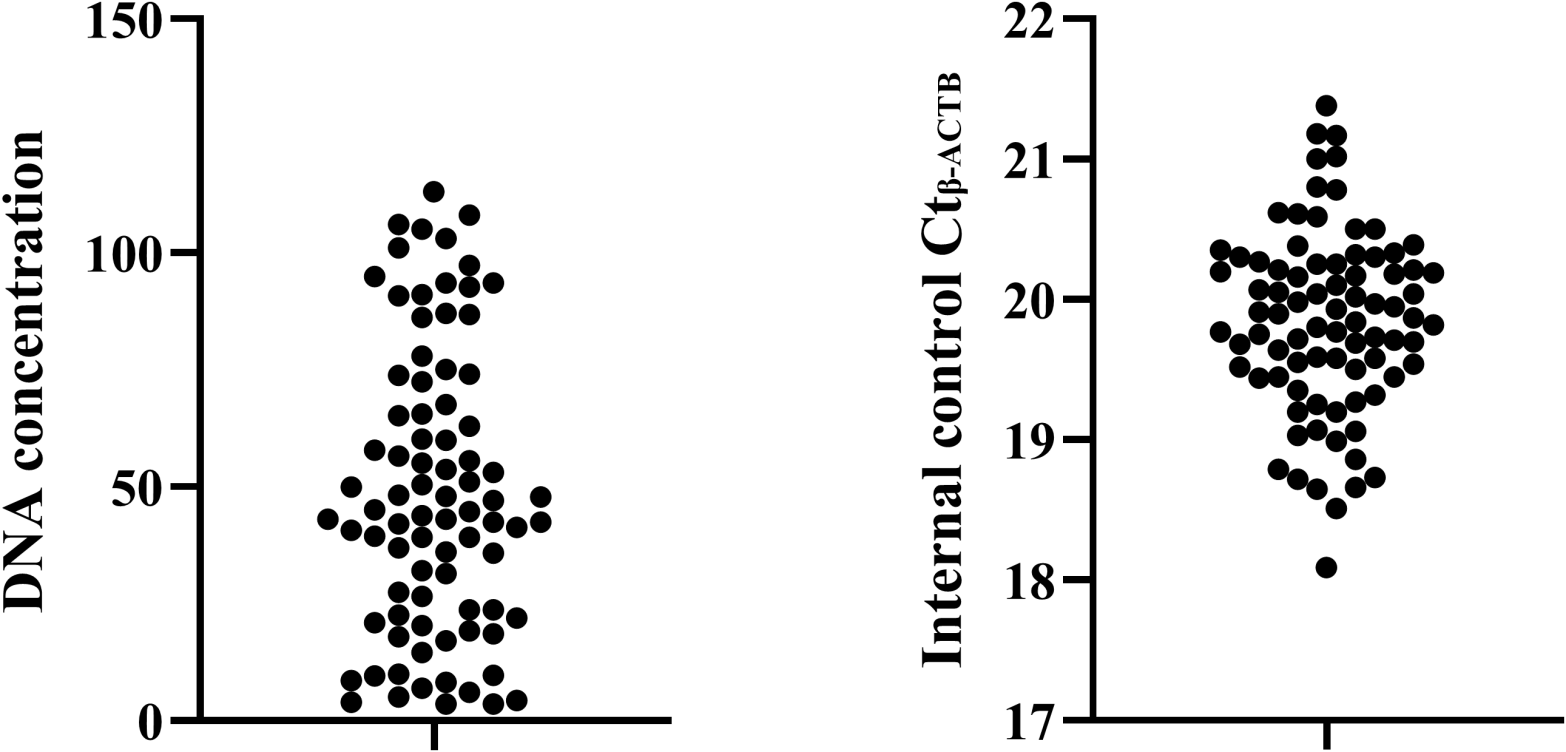
Quality control of methylation detection in TBNA needle tract washing fluid.

There are few studies on methylation detection of bronchoscopic TBNA needle tract washing fluid. To ensure the rigor of the study, this study sorted out the quality control of the detection results of needle tract washing fluid (**Figure 2**). When the DNA concentration is greater than 1ng/L, the internal reference Ct value can be controlled between 18-23, which meets the requirements of the detection product manual. All 83 needle tract washing fluid samples enrolled had DNA concentration greater than 1ng/L, and the internal reference Ct value was between 18.09-21.38, which fully met the quality control requirements, indicating that the methylation detection results were accurate and reliable.

### Setting of cut-off value for methylation detection in TBNA needle tract washing fluid

**Figure 3:**
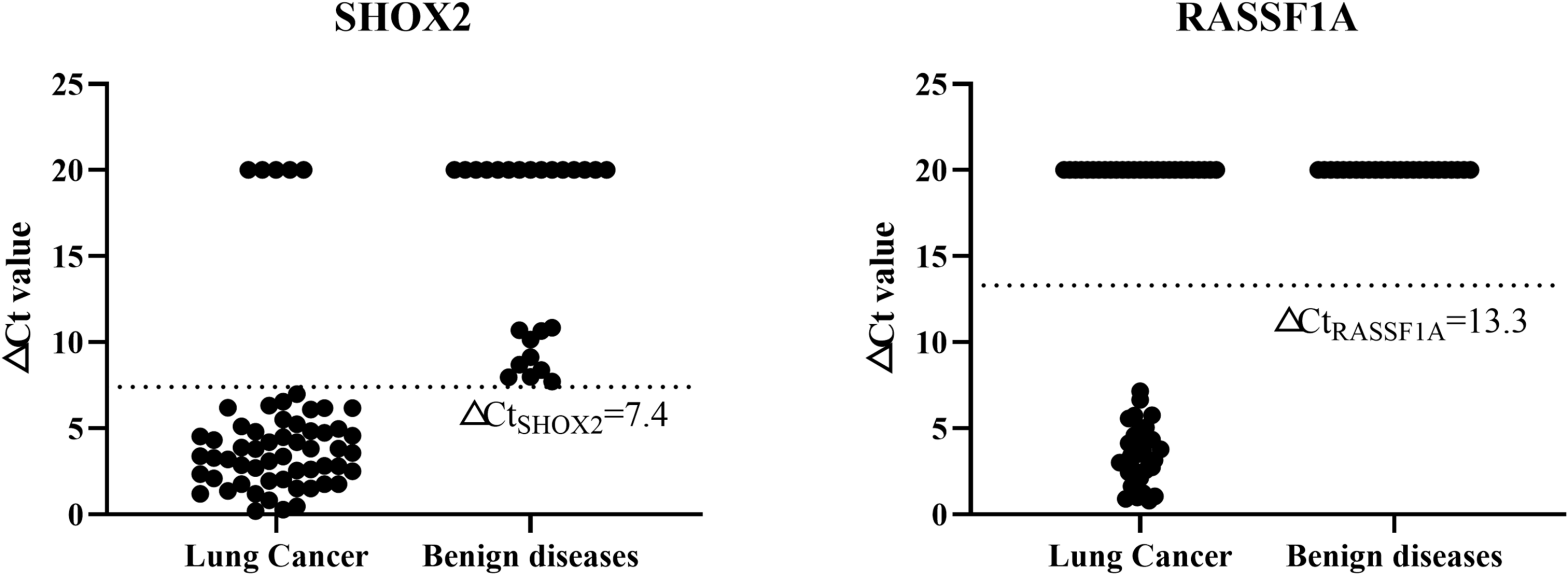
Setting of cut-off value for methylation detection in TBNA needle tract washing fluid.

The methylation detection reagent used in this study has NMPA-approved indications including alveolar lavage fluid, so for methylation detection of alveolar lavage fluid, the NMPA-recommended Cut-off value can be directly used, which is △Ct_SHOX2_ = 9, △Ct_RASSF1A_=12. However, the approved indications do not include TBNA needle tract washing fluid, so this study established the Cut-off value for methylation detection of needle tract washing fluid through ROC analysis. The samples were stratified and assigned into a training set (44 cases) and a verification set (39 cases). ROC analysis was performed using the training set to calculate Youden index. When the Youden index was the largest, the sensitivity of SHOX2 gene methylation detection was 86.7%, the specificity was 100%, and the corresponding Cut-off value was △Ct_SHOX2_ = 7.4. Similarly, when the Youden index was the largest, the sensitivity of RASSF1A gene methylation detection was 56.7%, the specificity was 100%, and the corresponding Cut-off value was △Ct_RASSF1A_=13.3. Substituting the set Cut-off value into the verification set, the sensitivities of SHOX2 and RASSF1A were 96.4% and 50.0% respectively, and the specificities were 100%, with no significant difference from the training set (P > 0.05). In the subsequent statistical analysis of needle tract washing fluid samples, △Ct_SHOX2_ = 7.4 and △Ct_RASSF1A_=13.3 were used as Cut-off values (**Figure 3**).

### Diagnostic sensitivity of gene methylation detection and morphology-based pathology

The diagnostic sensitivity was calculated for samples of different sample types and pathological types. The results showed (**Tables 2**, **3**) that for alveolar lavage fluid samples, the sensitivities of SHOX2 and RASSF1A methylation detection were 91.4% and 49.1% respectively, and the sensitivity of combined detection of the two was 94.1% (95% CI 0.9015 – 0.9651, PPV = 100%), which was significantly higher than 30.0% (95% CI 0.2433 – 0.3636, PPV = 100%) of morphological pathological diagnosis (χ^2^ = 135.2, P < 0.001). Among them, 144 patients were positive for methylation detection and negative for cytological diagnosis, and 3 patients were negative for methylation detection and positive for cytological diagnosis. For different pathological types, the sensitivity of combined detection of SHOX2 and RASSF1A was higher than that of a single index, and was always significantly higher than that of morphological pathological diagnosis (P < 0.001).

**Table 2:** Sensitivity of different diagnostic methods for bronchoalveolar lavage fluid.

	Total	SHOX2		RASSF1A		SHOX2+RASSF1A		Morphology-based pathology	
		n	Sensitivity	n	Sensitivity	n	Sensitivity	n	Sensitivity
Lung cancer	220	201	91.4%	108	49.1%	207	94.1%	66	30.0%
Lung adenocarcinoma	38	32	84.2%	17	44.7%	32	84.2%	13	34.2%
Lung squamous cell carcinoma	112	108	96.4%	39	34.8%	110	98.2%	35	31.3%
Small cell lung cancer	38	31	81.6%	31	81.6%	33	86.8%	12	31.6%
Unclassified lung cancer	32	30	93.8%	21	65.6%	32	100.0%	6	18.8%

**Table 3:** Sensitivity of different diagnostic methods for TBNA biopsy.

	Total	SHOX2		RASSF1A		SHOX2+RASSF1A		Morphology-based pathology	
		n	Sensitivity	n	Sensitivity	n	Sensitivity	n	Sensitivity
Lung cancer	58	53	91.4%	31	53.4%	55	94.8%	50	86.2%
Lung adenocarcinoma	39	34	87.2%	19	48.7%	34	87.2%	33	84.6%
Lung squamous cell carcinoma	15	15	100.0%	8	53.3%	15	100.0%	13	86.7%
Small cell lung cancer	4	4	100.0%	4	100.0%	4	100.0%	4	100.0%
Unclassified lung cancer	0	–	–	–	–	–	–	–	–

For TBNA needle tract washing fluid samples, the sensitivity of combined methylation detection was 94.8% (95% CI 0.8586 – 0.9823, PPV = 100%), which was higher than that of morphological pathological diagnosis of TBNA samples (86.2%, 95% CI 0.7507 – 0.9284, PPV = 100%), but there was no significant difference between the two (χ^2^ = 1.8, P = 0.182). Among them, 7 patients were positive for methylation detection and negative for histopathological diagnosis, and 2 patients were negative for methylation detection and positive for histopathological diagnosis. All these 7 patients were diagnosed with lung cancer in subsequent surgical treatment, who represented the net benefit population of methylation detection. For samples of patients with lung adenocarcinoma and lung squamous cell carcinoma, higher sensitivity of methylation detection was also observed, but the difference was not significant (P > 0.05).

It is worth noting that the overall sensitivity of TBNA morphological pathological diagnosis was 86.2%, while that of alveolar lavage fluid morphological pathological diagnosis was only 30.0%, and the difference was statistically significant (P < 0.001).

### Relationship between pathological stage and diagnostic sensitivity

In alveolar lavage fluid and TBNA needle tract washing fluid samples, the diagnostic sensitivity of combined detection of SHOX2 and RASSF1A methylation in stage I patients was 81.8% and 66.7% respectively, which was significantly lower than that in stage II-IV patients (P < 0.05).

In samples of each pathological stage, the sensitivity of alveolar lavage fluid methylation detection was significantly higher than that of morphological pathological diagnosis. Even for patients with advanced lung cancer, the sensitivity of morphological pathological diagnosis of alveolar lavage fluid was only 24.7-33.8%, which could not meet the needs of clinical diagnosis at all. The sensitivity of morphological pathological diagnosis of TBNA samples was greatly improved. The detection sensitivity of samples from stage I and III patients was slightly lower than that of methylation detection (66.7% VS 50.0%, 100% VS 80.0%). The detection sensitivity of stage II and IV samples was consistent with that of methylation detection (**Table 4**).

**Table 4:** Comparison of sensitivity of SHOX2 + RASSF1A detection and morphology-based pathology detection in BALF and TBNA at different tumor stages.

Tumor stage	Bronchoalveolar lavage fluid					Transbronchial needle aspiration				
		SHOX2+RASSF1A		Morphology-based pathology			SHOX2+RASSF1A		Morphology-based pathology	
	Total	n	Sensitivity	n	Sensitivity	Total	n	Sensitivity	n	Sensitivity
Stage I	11	9	81.8%	3	27.3%	6	4	66.7%	3	50.0%
Stage II	27	26	96.3%	13	48.1%	4	4	100.0%	4	100.0%
Stage III	73	70	95.9%	18	24.7%	15	15	100.0%	12	80.0%
Stage IV	77	70	90.9%	26	33.8%	33	31	93.9%	31	93.9%

### ROC analysis of different diagnostic methods

**Figure 4:**
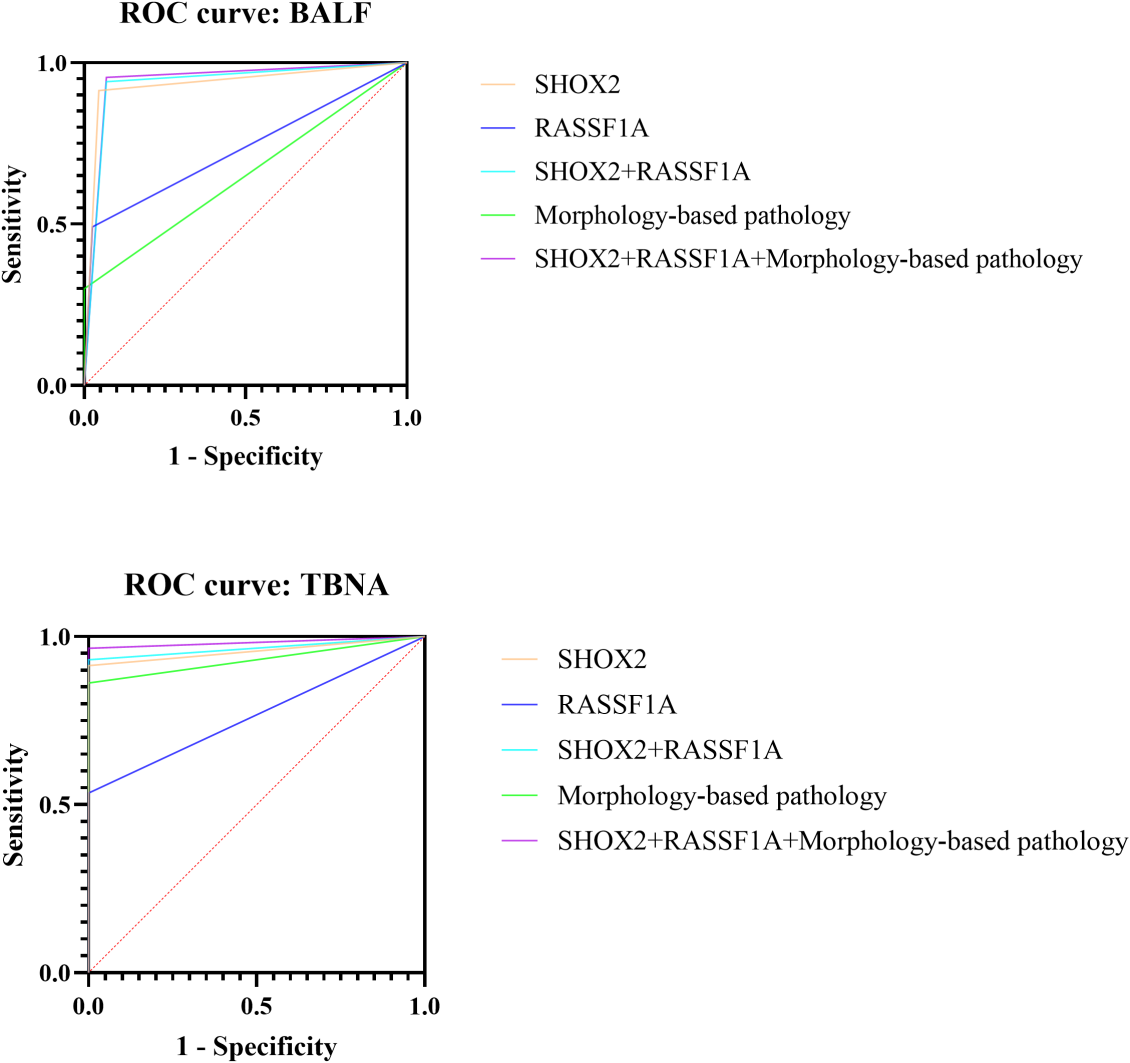
Diagnostic efficacy of different diagnostic methods in bronchoalveolar lavage fluid and TBNA samples.

It can be seen from **Table 5**; **Figure 4** that for alveolar lavage fluid samples, the sensitivity of combined detection of SHOX2 and RASSF1A gene methylation was 94.1%, the specificity was 93.2%, and the AUC value was 0.9363 (95% CI 0.9127-0.9599), which was higher than any single index. The specificity of morphology-based pathology was 100%, but the sensitivity was only 30.0%, and the AUC value was 0.6500. When gene methylation detection was combined with morphology-based pathology to issue a joint diagnosis report, the AUC value was further increased to 0.9431, and the clinical application value was significant.

**Table 5:** Diagnostic performance of SHOX2, RASSF1A, and morphology-based pathology in BALF and TBNA.

	AUC					
	Value	95% CI	Sensitivity	Specificity	PPV	NPV
Bronchoalveolar lavage fluid						
SHOX2	0.9340	0.9091 - 0.9590	91.4%	95.4%	94.3%	93.3%
RASSF1A	0.7326	0.6868 - 0.7785	49.1%	97.4%	91.7%	78.6%
SHOX2+RASSF1A	0.9363	0.9127 - 0.9599	94.1%	93.2%	91.5%	95.1%
Morphology-based pathology	0.6500	0.6012 - 0.6988	30.0%	100.0%	100.0%	68.4%
SHOX2+RASSF1A+Morphology-based pathology	0.9431	0.9210 - 0.9652	95.5%	93.2%	91.8%	96.1%
Transbronchial needle aspiration						
SHOX2	0.9569	0.9132 - 1.0000	91.4%	100.0%	100.0%	78.1%
RASSF1A	0.7672	0.6692 - 0.8653	53.5%	100.0%	100.0%	47.2%
SHOX2+RASSF1A	0.9655	0.9263 - 1.0000	93.1%	100.0%	100.0%	86.2%
Morphology-based pathology	0.9310	0.8760 - 0.9861	86.2%	100.0%	100.0%	76.9%
SHOX2+RASSF1A+Morphology-based pathology	0.9828	0.9550 - 1.0000	96.6%	100.0%	100.0%	92.6%

For TBNA and needle tract washing fluid samples, the sensitivity of combined methylation detection was 93.1%, the specificity was 100%, and the AUC value was 0.9655 (95% CI 0.9263-1.0000). After combining methylation detection with morphology-based pathology, the sensitivity was increased to 96.6%, the AUC value was 0.9828, the positive predictive value reached 100%, and the negative predictive value reached 92.6%.

For alveolar lavage fluid and TBNA samples, gene methylation detection combined with morphological pathological diagnosis is a good combination scheme to make up for missed diagnosis.

### Clinical value of strongly positive detection results

In order to strengthen clinical cooperation with thoracic surgery, in actual clinical use, we defined a strongly positive result as △Ct being 3 less than the Cut-off value. Specifically, in alveolar lavage fluid, the positive and negative Cut-off values were set as △Ct_SHOX2_ < 9 and △Ct_RASSF1A_ < 12. 6 ≤ △Ct_SHOX2_ < 9 and 9 ≤ △Ct_RASSF1A_ < 12 were defined as weakly positive, and △Ct_SHOX2_ < 6 and △Ct_RASSF1A_ < 9 were defined as strongly positive. In TBNA needle tract washing fluid, the positive and negative Cut-off values were set as △Ct_SHOX2_ < 7.4 and △Ct_RASSF1A_ < 13.3. 4.4 ≤ △Ct_SHOX2_ < 7.4 and 10.3 ≤ △Ct_RASSF1A_ < 13.3 were defined as weakly positive, and △Ct_SHOX2_ < 4.4 and △Ct_RASSF1A_ < 10.3 were defined as strongly positive.

**Figure 5:**
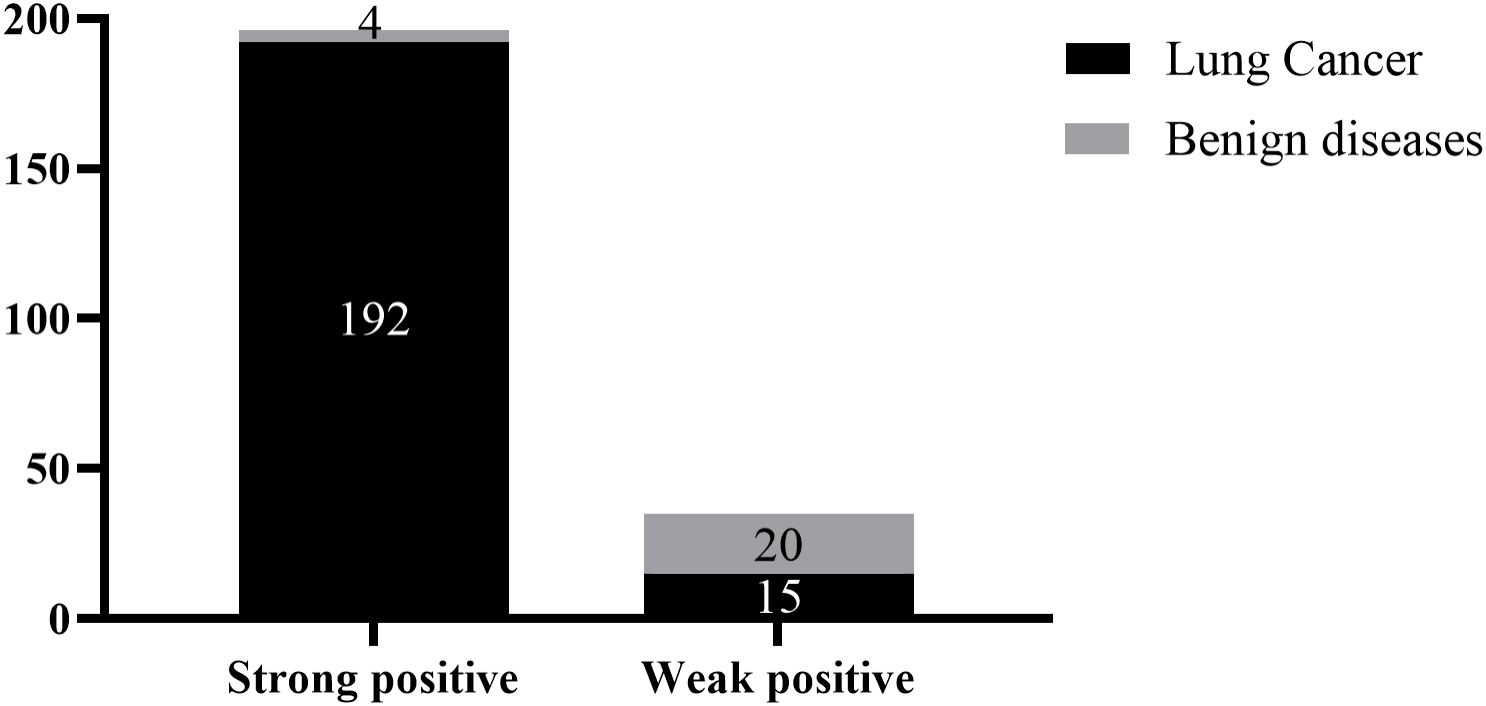
Positive predictive value of strongly positive results in methylation detection.

This study further analyzed the impact of positive and strongly positive results on the diagnostic accuracy of patients (**Figure 5**). Among the alveolar lavage fluid samples of lung cancer patients, 192 cases were strongly positive in methylation detection and 15 cases were weakly positive; Among the alveolar lavage fluid of patients with benign diseases, 15 cases were strongly positive in methylation detection and 20 cases were weakly positive. The positive predictive value of strongly positive results was 98.0%, and that of weakly positive results was 42.9%, with a significant difference between the two (2 = 113.7, P < 0.001). For TBNA needle tract washing fluid samples, the positive predictive values of both results were 100%.

### Follow-up survey of patients with undetermined diagnosis

**Figure 6:**
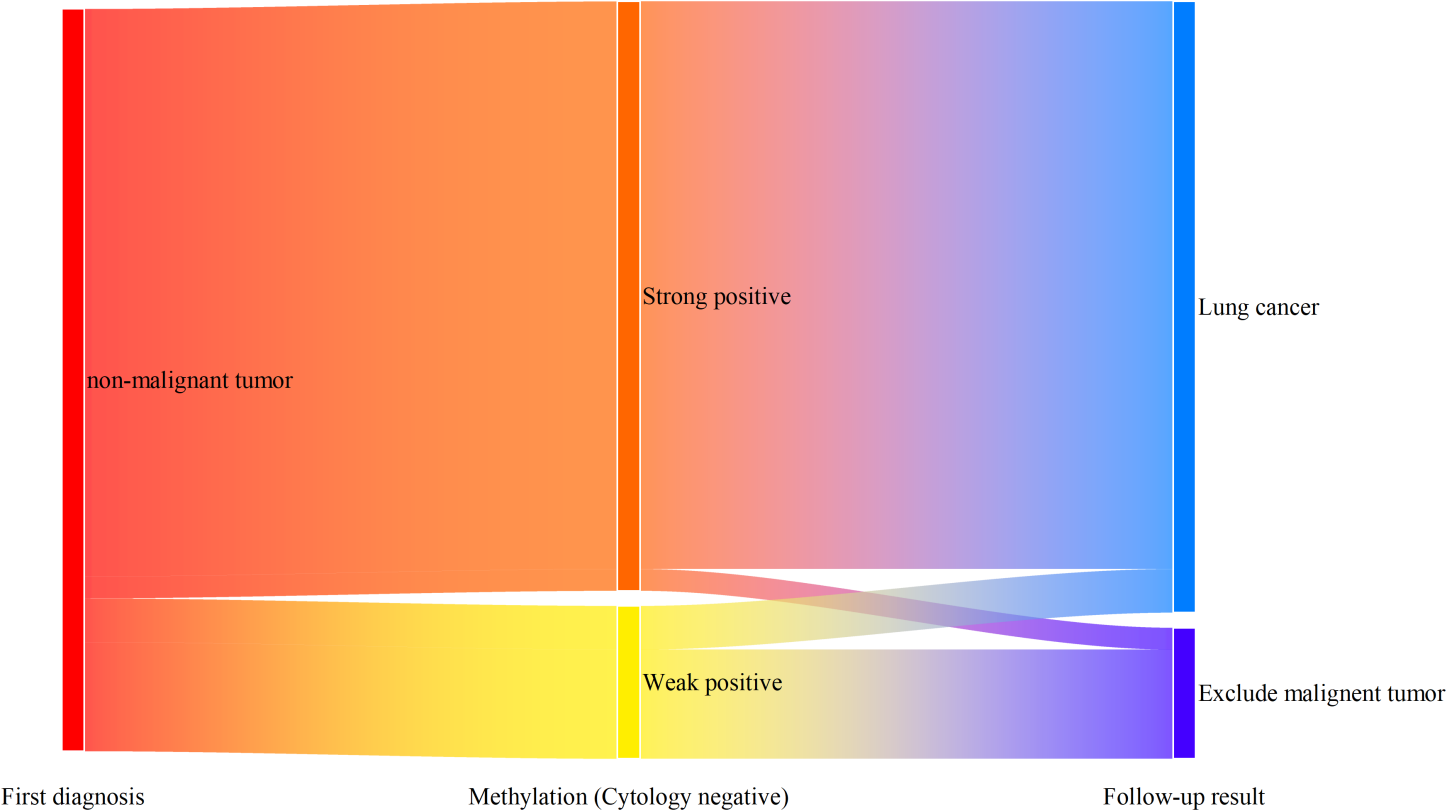
Follow-up of methylation-positive patients with initial diagnosis of non-malignant tumors.

Among the 220 alveolar lavage fluid samples enrolled in the study, 34 cases were positive in combined methylation detection (including weakly positive and strongly positive) at the initial diagnosis. These 34 patients were followed up (**Figure 6**), with a median follow-up time of 18 months. The results showed that among 27 patients with strongly positive methylation detection, 26 were diagnosed with lung cancer in other hospitals, and only one was clearly excluded from lung cancer, with a diagnostic accuracy of 96.3%. Among 7 patients with weakly positive methylation detection results, 2 were diagnosed with lung cancer in other hospitals, and 5 were excluded from lung cancer, with a relatively low accuracy (28.6%).

## Discussion

As an invasive diagnostic method, bronchoscopic diagnosis is the last gateway for lung cancer diagnosis and sampling (15). In actual clinical work, the most common feeling of clinicians is that it is not easy to observe the lesion site through endoscope and take accurate samples, but traditional morphology-based pathology often cannot give an accurate diagnosis result. For lesions that cannot be observed by endoscope, the quality of distal lavage sampling is poor, and the missed diagnosis rate of morphology-based pathology is higher. The limitation of morphology-based pathology is due to it is an empirical discipline, which is greatly affected by subjective factors, especially for primary medical institutions lacking experience in pathological diagnosis (16). This forces medical practitioners to explore a diagnostic method based on objective numerical detection results, as a supplement and extension of morphology-based pathology.

The sampling quality of bronchoscopic TBNA is very high, but the sampling volume is small, which cannot meet the needs of other tests while doing morphology-based pathology. In China, the clinical charge for bronchoscopic TBNA is usually high, and patients subjectively hope that high-quality sampling methods can be matched with higher-quality testing schemes. The sampling volume of TBNA is small and cannot be directly used for gene methylation detection. We tried to wash the needle tract of the puncture needle and obtained the residual exfoliated cells in the needle tract. Encouragingly, the concentration of DNA extracted from these exfoliated cells all exceeded the recommended concentration of 1ng/L, which is sufficient for gene methylation detection. This also provides a technical basis for adding other gene detection methods to TBNA samples, in addition to morphology-based pathology testing. In addition, although TBNA needle tract washing fluid is essentially a type of exfoliated cell sample, it has not obtained NMPA indication approval, so this study re-determined the Cut-off value through ROC analysis.

Among the 278 lung cancer patients enrolled in this study, there were 77 cases of lung adenocarcinoma, 127 cases of lung squamous cell carcinoma, 42 cases of small cell lung cancer, and 32 cases of unclassified lung cancer. Among them, the proportion of patients with lung squamous cell carcinoma was the highest, reaching 45.7%, which was significantly higher than other pathological types (P < 0.05). According to epidemiological statistics, lung adenocarcinoma is the most common pathological type (17). The large number of patients with lung squamous cell carcinoma in this study is mainly because after imaging evaluation, clinicians will recommend patients to undergo bronchoscopy only if the lesion can be reached by bronchoscopy. Most of these patients’ lesions are in the airway or close to the proximal bronchus. We will first brush with a brush and then wash with normal saline. The lesions damaged by brushing will release more exfoliated cells during normal saline washing, improving the detection rate of cancer cells. The lesions located in the distal bronchus or lung parenchyma are mostly adenocarcinoma, and we usually recommend patients to undergo percutaneous puncture biopsy to obtain higher-quality diagnostic samples.

When analyzing the diagnostic sensitivity of methylation detection, we found that the diagnostic sensitivity of different indicators varies greatly, and the sensitivity of dual-gene combined diagnosis is significantly higher than that of a single gene (P < 0.05), which is similar to the research results of Zhang et al. (18), indicating that the two selected genes have strong complementarity. The sensitivity and AUC value of gene methylation detection are significantly higher than those of morphological pathological diagnosis (P < 0.05), which strongly suggests that clinicians should match gene methylation detection when patients undergo bronchoscopy for the first time. Provide clinicians with rich diagnostic information from two different aspects of morphology-based pathology and genes, improve the success rate and accuracy of patients’ first bronchoscopy, avoid patients having to undergo bronchoscopy again due to pathological missed diagnosis, and reduce patients’ economic burden and psychological pressure.

In this study, the sensitivity of morphological pathological diagnosis of alveolar lavage fluid was only 30.0%. We know that usually the quality of distal lavage sampling is poor, and the accuracy of pathological diagnosis is not high. Even if we adopt the mode of first brushing and then washing, the diagnostic effectiveness of morphology-based pathology is still unsatisfactory, which also truly reflects the dilemma of pathological diagnosis in our primary hospital. It also strengthens our determination to seek more diagnostic methods besides morphology-based pathology. From the detection results of TBNA samples, although methylation detection has higher sensitivity than morphology-based pathology, there is no significant improvement in AUC value (P > 0.05). But we believe that from the perspective of patient psychology, patients who can afford bronchoscopic TBNA examination must hope to obtain a more comprehensive and accurate disease assessment. The accurate lesion sampling method is matched with the primary diagnostic method, which is essentially a waste of medical resources. This was also demonstrated by the 7 patients with net benefits from methylation detection identified in the present study. Although this single-center study lacked external validation and the data may not be sufficiently compelling, it indicates a promising direction and trend for further research. Therefore, we more recommend patients to add gene methylation detection or other gene detection schemes on the basis of morphology-based pathology.

When analyzing the relationship between patients’ pathological stage and methylation detection sensitivity, we found that for the two different sample types, the diagnostic sensitivity of stage I patients was significantly lower than that of stage II-IV patients (P < 0.05). This is because the tumor volume of stage I patients is generally small, the number of tumor cells collected in alveolar lavage fluid is small, and TBNA biopsy may not accurately obtain the tumor site, resulting in too low proportion of malignant tumor cells in the sample (19). Studies have shown that during gene methylation detection, more than 250 copies of tumor genes in the sample can ensure accurate detection of gene methylation signals (20). Although there are certain requirements for the sampling quality of early patients, the diagnostic effectiveness of gene methylation detection is still significantly higher than that of morphological pathological diagnosis.

Strongly positive detection results are new empirical diagnostic criteria summarized based on a large number of previous clinical practices. Initially, we hoped to define a strongly positive result as a tenfold increase in the concentration of methylated genes in the original sample, but in actual work, it was found that it is difficult to correspond this standard to the △Ct value output by the PCR instrument. Therefore, we defined a strongly positive result as △Ct being 3 less than the Cut-off value. In fact, at this time, the concentration of methylated genes in the original sample increased by 2 to the power of 3 times (about 8 times), which is close to our initial assumption. The reason for setting the diagnostic standard for strongly positive is that we hope to have better interaction with surgeons in the interpretation of gene detection results. In the process of patient transfer, these expensive diagnostic results can play a role in treatment decisions in different clinical departments. Data show that the positive predictive value of strongly positive gene methylation detection for lung cancer is as high as 98.0%, which can be used as an independent clinical indication to guide surgical decision-making. In addition, in the statistics of follow-up patients, it was found that 96.3% of patients with strongly positive gene methylation in alveolar lavage fluid were diagnosed with lung cancer in subsequent detection and diagnosis in other hospitals. It further proves the early warning value of strongly positive detection results. Clinicians should pay special attention when finding patients with strongly positive results, and try to improve the examination to avoid patient loss.

This study also has limitations. First, it is a retrospective study, mainly including patients whose lesions can be reached by bronchoscopy as judged by imaging. There are few enrolled patients with peripheral lung cancer, so it cannot cover all lung cancer groups, and the data analysis results may be biased. For TBNA samples, the nature of the single-center study combined with the limited sample size precluded valid external validation, which may have led to an over-optimistic interpretation of the results. In future research, a multicenter study design should be adopted to further validate the application value of methylation detection in the auxiliary diagnosis of lung cancer. This study did not combine gene methylation detection results with patient treatment evaluation. Studies have shown that patients with positive gene methylation may be more sensitive to chemotherapy (21). The combination of some new demethylation drugs and chemotherapy may improve the efficacy (22). In future studies, we will consider introducing more innovative diagnostic methods, combined with methylation detection and morphology-based pathology, to establish a new model for lung cancer diagnosis through logistic regression or machine learning.

## Conclusions

In conclusion, this study innovatively proposed a methylation detection scheme for alveolar lavage fluid and bronchoscopic TBNA needle tract washing fluid, and combined with morphology-based pathology to improve diagnostic effectiveness. The results showed that the sensitivity of alveolar lavage fluid methylation detection was 94.1% and the specificity was 93.2%; The sensitivity of TBNA needle tract washing fluid methylation detection was 93.1% and the specificity was 100%. After combining with morphology-based pathology, the AUC values reached 0.9431 (95% CI: 0.9210 - 0.9652) and 0.9828 (95% CI: 0.9550 - 1.0000) respectively. While providing more diagnostic and therapeutic basis for clinicians, it improves the success rate and accuracy of patients’ first bronchoscopy.

## Data Availability

The original contributions presented in the study are included in the article/supplementary material. Further inquiries can be directed to the corresponding authors.
